# Pharmacoeconomic analysis of adjuvant oral capecitabine *vs* intravenous 5-FU/LV in Dukes' C colon cancer: the X-ACT trial

**DOI:** 10.1038/sj.bjc.6603059

**Published:** 2006-04-18

**Authors:** J Cassidy, J-Y Douillard, C Twelves, J J McKendrick, W Scheithauer, I Bustová, P G Johnston, K Lesniewski-Kmak, S Jelic, G Fountzilas, F Coxon, E Díaz-Rubio, T S Maughan, A Malzyner, O Bertetto, A Beham, A Figer, P Dufour, K K Patel, W Cowell, L P Garrison

**Affiliations:** 1 Cancer Research UK, Department of Medical Oncology, University of Glasgow, Garscube Estate, Bearsden, Glasgow G61 1DB, UK; 2 Centre René Gauducheau, Site Hospitalier Nord, Bld J Monod, 44805 Saint Herblain, Nantes, France; 3 Tom Connors Cancer Research Centre, University of Bradford, Richmond Road, Bradford BD7 1DP, UK; 4 Department of Oncology, Box Hill Hospital, Nelson Road, Melbourne, Victoria, Australia; 5 Medizinische Universität Wien, Univ. Klinik für Innere Medizin I, Währinger Gürtel 18–20, A-1090 Vienna, Austria; 6 Nemocnice Ceske Budejovice, Klinika Onkolgie, Bozeny Nemcove 54, 370 87 C Budejovice, Czech Republic; 7 Department of Oncology, Centre for Cancer Research, Queen's University Belfast, Belfast BT9 7AB, UK; 8 PCK Maritime Hospital, Medical Oncology and Radiotherapy Department, ul Powstania Styczniowego 1, 81–519 Gdynia, Poland; 9 Institute for Oncology and Radiology of Serbia, Pasterova 14, 11000 Belgrade, Serbia and Montenegro; 10 Department of Medical Oncology, Papageorgiou Hospital, Aristotle University of Thessaloniki School of Medicine, Thessaloniki, Greece; 11 Northern Centre for Cancer Treatment, Newcastle General Hospital, Westgate Road, Newcastle-upon-Tyne NE4 6BE, UK; 12 Hospital Universitario San Carlos, Plaza Cristo Rey S/N, Madrid 28040, Spain; 13 Velindre Hospital, Whitchurch, Cardiff CF4 7XL, UK; 14 Clinica de Oncologia Medica S/C Ltda., Av. Nove de Julho, 4644, Sao Paulo 01406-100, Brazil; 15 COES, Ospedale S Giovanni Battista sede Molinette, Corso Bramante, 88, 12126 Torino, Italy; 16 Klinik und Poliklinik für Chirurgie Klinikum der Universität Regensberg, Franz-Josef Strauss Allee 11, 93053 Regensberg, Germany; 17 Oncology Institute, the Elias Sourasky Medical Center, Tel-Aviv 64239, Israel; 18 Centre de Lutte Contre le Cancer Paul-Strauss, 3 rue de la Porte de l'Hôpital, F-67065 Strasbourg, France; 19 Hoffmann-La Roche Inc., Nutley, NJ, USA; 20 Roche UK, Welwyn Garden City, UK; 21 University of Washington, Seattle, Washington 98195-7630, USA

**Keywords:** capecitabine, 5-fluorouracil/leucovorin, adjuvant, colon cancer, pharmacoeconomics, cost-effectiveness

## Abstract

Oral capecitabine (Xeloda®) is an effective drug with favourable safety in adjuvant and metastatic colorectal cancer. Oxaliplatin-based therapy is becoming standard for Dukes' C colon cancer in patients suitable for combination therapy, but is not yet approved by the UK National Institute for Health and Clinical Excellence (NICE) in the adjuvant setting. Adjuvant capecitabine is at least as effective as 5-fluorouracil/leucovorin (5-FU/LV), with significant superiority in relapse-free survival and a trend towards improved disease-free and overall survival. We assessed the cost-effectiveness of adjuvant capecitabine from payer (UK National Health Service (NHS)) and societal perspectives. We used clinical trial data and published sources to estimate incremental direct and societal costs and gains in quality-adjusted life months (QALMs). Acquisition costs were higher for capecitabine than 5-FU/LV, but higher 5-FU/LV administration costs resulted in 57% lower chemotherapy costs for capecitabine. Capecitabine *vs* 5-FU/LV-associated adverse events required fewer medications and hospitalisations (cost savings £3653). Societal costs, including patient travel/time costs, were reduced by >75% with capecitabine *vs* 5-FU/LV (cost savings £1318), with lifetime gain in QALMs of 9 months. Medical resource utilisation is significantly decreased with capecitabine *vs* 5-FU/LV, with cost savings to the NHS and society. Capecitabine is also projected to increase life expectancy *vs* 5-FU/LV. Cost savings and better outcomes make capecitabine a preferred adjuvant therapy for Dukes' C colon cancer. This pharmacoeconomic analysis strongly supports replacing 5-FU/LV with capecitabine in the adjuvant treatment of colon cancer in the UK.

The current global standard adjuvant treatment for Dukes' C (stage III) colon cancer is intravenous (i.v.) administration of bolus 5-fluorouracil (5-FU) and leucovorin (LV), either weekly or monthly, over a period of 6–8 months (Van Cutsem *et al*, 2002). Adjuvant 5-FU/LV reduces the risk of relapse and prolongs survival in patients with resected colon cancer (IMPACT, 1995; O'Connell *et al*, 1997; Haller *et al*, 1998; Wolmark *et al*, 1999; Porschen *et al*, 2001; Arkenau *et al*, 2003).

Although the clinical benefits associated with adjuvant 5-FU/LV are significant, it is clear that more effective, convenient and better-tolerated treatments are required. Capecitabine (Xeloda®, F Hoffmann-La Roche, Basel, Switzerland) is a convenient oral fluoropyrimidine that generates 5-FU preferentially in tumour tissue through a three-step enzymatic cascade (Miwa *et al*, 1998). As first-line therapy for metastatic colorectal cancer, oral capecitabine achieved improved response rates (26 *vs* 17%, respectively), and equivalent progression-free and overall survival compared with monthly bolus i.v. 5-FU/LV (Van Cutsem *et al*, 2004). Capecitabine was also better tolerated than 5-FU/LV and its administration was associated with a reduced consumption of medical resources (Twelves *et al*, 2001). These results led to the approval of capecitabine in 2001 as a first-line alternative to 5-FU/LV in metastatic colorectal cancer.

The effectiveness of capecitabine in the metastatic setting provided a rationale for its use as adjuvant therapy for colon cancer. A large, randomised phase III study (X-ACT) was undertaken to compare the efficacy and tolerability of adjuvant oral capecitabine *vs* bolus i.v. 5-FU/LV (Mayo Clinic regimen) over 24 weeks in 1987 patients with Dukes' C colon cancer (Twelves *et al*, 2005). This study demonstrated that capecitabine is at least as effective as 5-FU/LV with significant superiority in relapse-free survival (*P*=0.0407) and a trend towards improved disease-free (*P*=0.0528) and overall survival (*P*=0.0706). In addition, an improved safety profile was noted in favour of capecitabine (Scheithauer *et al*, 2003; Twelves *et al*, 2005).

On 31 March 2005, capecitabine received approval for the adjuvant treatment of Dukes' C colon cancer from the European Medicines Agency (EMEA) Committee for Medicinal Products for Human Use (CHMP). Capecitabine was also recently approved as a single agent for the adjuvant treatment of Dukes' C colon cancer by the US Food and Drug Administration (FDA) in patients ‘who have undergone complete resection of the primary tumour, when treatment with fluoropyrimidine therapy alone is preferred’. Patients have long expressed a preference for oral fluoropyrimidine therapy instead of i.v. treatment (Liu *et al*, 1997; Borner *et al*, 2002) and oncologists in Europe and the US are now in a better position to satisfy this preference.

Clearly, the results of the X-ACT trial suggest that capecitabine can be used instead of 5-FU/LV in the adjuvant treatment of Dukes' C colon cancer, and we have seen that oral treatment is preferable from the point of view of most patients. However, with ever-increasing pressures to control medical costs, the decision of whether or not to use a treatment may not be based on clinical effectiveness alone. Medical guidelines and treatment decision-making increasingly give consideration to economic costs associated with achieving the health benefits of a therapy. The National Institute for Health and Clinical Excellence (NICE), for example, considers ‘how well the medicine or treatment works in relation to how much it costs the National Health Service (NHS)’ (NICE, 2005). These comparisons of cost-effectiveness can reveal the balance between costs and savings among alternative treatments and thereby assist healthcare providers in prioritising use of available medical resources to maximise health gain (Siegel *et al*, 1996; Weinstein *et al*, 1996). Using data collected prospectively during the X-ACT trial, we undertook this pharmacoeconomic analysis to evaluate the cost-effectiveness of adjuvant capecitabine *vs* standard adjuvant therapy (bolus 5-FU/LV (Mayo Clinic regimen)) in patients with Dukes' C colon cancer, from the UK NHS perspective, as well as from a societal perspective.

## PATIENTS AND METHODS

Medical resource use and cost-effectiveness analyses were conducted as part of a prospective pharmacoeconomic evaluation of the X-ACT study. In brief, the X-ACT study was an open-label, multinational, randomised, phase III trial of adjuvant therapy for resected, histologically confirmed Dukes' C colon carcinoma (Scheithauer *et al*, 2003; Twelves *et al*, 2005). Patients were randomised to 24 weeks' treatment with either eight cycles of oral capecitabine 1250 mg m^−2^ twice daily, days 1–14 every 21 days (*n*=1004), or six cycles of rapid-infusion i.v. leucovorin 20 mg m^−2^ followed immediately by i.v. bolus 5-FU 425 mg m^−2^, days 1–5 every 28 days (Mayo Clinic regimen) (*n*=983).

### Design and structure of the pharmacoeconomic model

A health-state transition model was developed to assess healthcare costs, quality-adjusted survival and overall cost-effectiveness of capecitabine compared with 5-FU/LV. The model consists of three health states – stable (prerelapse; disease- and relapse-free), postrelapse and death, with further subclassification of the postrelapse category into relapse (i.e. during subsequent treatment for metastatic colon cancer), remission and the 12-month period before death. A relapse event was classified as instances of relapse, new colon cancer or death due to colon cancer or treatment. These health states allowed us, in effect, to partition overall survival into pre- and postrelapse periods, using the relapse-free and overall survival data for capecitabine and 5-FU/LV observed in the X-ACT clinical trial. The model incorporates costs during chemotherapy using the medical resource utilisation data collected during the X-ACT clinical trial. In addition, possible outcomes for postchemotherapy costs were considered for the postrelapse health states.

The time spent in each health state was estimated by extrapolating the relapse-free and overall survival follow-up data from the X-ACT clinical trial to a lifetime horizon. Health outcomes were measured as life months (LMs) gained and quality-adjusted life months (QALMs) gained, where QALMs are a measure of the time spent in each health state, weighted by the quality of life (utility) in that health state. The incremental cost-effectiveness ratio, measured as the cost per QALM gained, was estimated by dividing the difference in total costs in each arm by the increase in survival for treatment with capecitabine compared with 5-FU/LV. Technically, a meaningful incremental cost-effectiveness ratio cannot be calculated if a therapy being evaluated is found to be cost saving or cost neutral (i.e. negative or zero numerator) and either more or equally effective (i.e. positive or zero denominator). When a therapy is cost saving and more effective, it is termed ‘dominant’ because it is clearly preferred.

### Medical resource use and costs

The unit costs for medical resource utilisation during treatment are detailed in Table 1. Safety and medical resource use data were collected prospectively during the X-ACT clinical trial, throughout treatment and for 28 days after the last intake of study drug. Data were recorded at all study centres on case report forms. Data were collected on study drug administration (including cumulative dose, infusion duration and frequency), hospital admissions (including length of stay) and visits to providers and outpatient consultations for treatment-related adverse events (AEs). Consultations were categorised according to the type of healthcare provider (e.g. general practitioner, specialist or allied health professional) and location (e.g. emergency unit, home visit or clinic visit). It was assumed, based on expert opinion, that 5% of patient visits to hospital would have required ambulance transportation.

Chemotherapy drug costs were taken from the Monthly Index of Medical Specialties (September 2004), with costs for consultations, hospitalisations, accident and emergency care and ambulance transportation derived from *Unit Costs of Health and Social Care* published by the UK-based Personal Social Services Research Unit (Netten and Curtis, 2004). Cost of i.v. administration was taken from the UK Department of Health National Tariff (DOH, 2005). In the UK, patients receiving capecitabine see a specialist for a consultation and patients treated with 5-FU/LV go to an outpatient clinic in a hospital for i.v. administration. In addition, patients receiving 5-FU/LV will see a specialist during some of their drug administration visits. In the base case, it was assumed that 5-FU/LV patients would see a specialist for the same number of visits as patients receiving capecitabine, in addition to going to the outpatient clinic for i.v. administration.

The model also considered drugs used in the management of treatment-related AEs; the selection of drugs to be included in the model was based on expert clinical/pharmacist judgement. Within a class of drugs, the drug most commonly used in the clinical trial was used to estimate the unit cost in that class. The total cost of each medication was calculated by multiplying the daily cost of treatment by the total number of days of treatment used in each arm. This was then divided by the number of patients in the relevant treatment arm to provide the mean cost per patient.

Assumptions for the post-treatment costs were based on previously published lifetime costs of colorectal cancer (Etzioni *et al*, 2001; Ramsey *et al*, 2002). Costs associated with relapse were based on assumptions derived from a study reporting the cost-effectiveness of oxaliplatin/5-FU/LV in the adjuvant treatment of colorectal cancer (Aballéa *et al*, 2005). For the base case, the assumptions were: £100 monthly maintenance cost during prerelapse, £25 000 average cost during the relapse period, £200 monthly maintenance cost during postrelapse; and £10 000 average cost during the last 12 months of life.

### Societal costs

From a societal perspective, the model also considered indirect costs borne by the patient, such as cost of travel and time for outpatient and drug administration visits. Time assumptions included travel time, as well as waiting and encounter time and was assumed to be 1.5 h for outpatient visits for management of AEs, 8 h for hospitalisations for management of AEs and 2 and 4 h, respectively, for capecitabine consultation and 5-FU/LV administration visits (Twelves, 2003). The value assigned to this time was £12 per hour based on average hourly compensation in the UK. This information, together with the number of outpatient, hospital and drug administration visits in each treatment arm was used to determine the mean number of hours per patient in each treatment arm and the cost of this time. For travel costs, a 30-mile round trip was assumed and was assigned a value of £0.23 per mile. This information, together with the number of outpatient, hospital and drug administration visits in each treatment arm was used to determine the total travel cost per patient in each treatment arm.

### Survival analysis

The time a patient spent in each health state was estimated using partitioned survival analysis of the trial data (intent-to-treat population), with projections beyond the trial period for 5, 10-year and lifetime horizons. In effect, this analysis estimates the area under the time-to-event curves at each horizon for relapse and overall survival, and then derives the postrelapse time by subtracting the former from the latter. These extrapolations were based on fitting a log-normal distribution to the relapse-free and overall survival data for the capecitabine and 5-FU/LV treatment groups. These data were used to determine the amount of time that the average patient would spend in the pre- and postrelapse health states.

### Quality of life (utility)

Utility values for the health states were derived from the published literature (Ramsey *et al*, 2000). For both arms, it was assumed that utility was 0.8 during chemotherapy and was 0.86 during the stable (prerelapse) health state. An overall average utility of 0.59 was assumed for the postrelapse health states.

### Discounting

Discounting for the time value of money was applied to both cost and outcomes, according to the guidelines issued by the NICE, in order to compare alternative future levels of costs and benefits. In this analysis, an annual discount rate of 1.5% was applied to benefits and an annual discount rate of 6.0% was applied to all costs.

### Sensitivity analyses

One-way and multi-way sensitivity analyses were performed to test the robustness of the model. The sensitivity analyses widely varied key assumptions in the model, including time horizon, key cost parameters (during treatment and post-treatment) and overall cost-effectiveness.

## RESULTS

From November 1998 to November 2001, a total of 1987 patients were enrolled into the X-ACT study at 164 centres worldwide. The capecitabine and 5-FU/LV treatment arms included 1004 and 983 patients, respectively, and the treatment arms were well balanced. The efficacy and safety results have been reported previously (Scheithauer *et al*, 2003; Twelves *et al*, 2005).

### Chemotherapy costs

**Figure 1:**
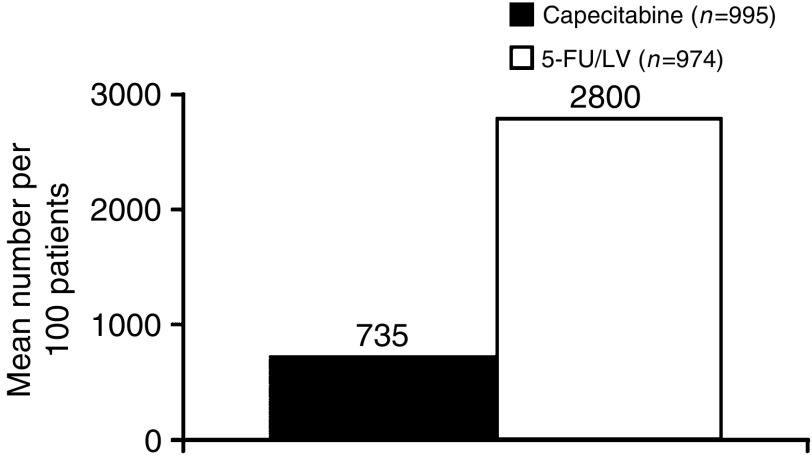
Number of treatment visits for chemotherapy administration or AEs with capecitabine *vs* 5-FU/LV.

Although the mean cost of chemotherapy drugs per patient was higher in the capecitabine arm (£2081 compared with £602 in the 5-FU/LV arm), the mean number of treatment administration visits was increased almost four-fold with the i.v. 5-FU/LV regimen (28 visits in 6 months) compared with capecitabine (7.4 visits in 6 months) (Figure 1). This resulted in increased costs for chemotherapy administration in the 5-FU/LV arm compared with the capecitabine treatment arm (£5151 and £419, respectively). Thus, considering both drugs and their administration, chemotherapy costs are lower by £3253 (57% lower) for capecitabine *vs* 5-FU/LV.

### Cost of managing AEs

The improved safety profile with capecitabine compared with 5-FU/LV was reflected in the need for fewer costly medications for the management of treatment-related AEs in the capecitabine treatment arm compared with 5-FU/LV (Table 2). In particular, capecitabine reduced the need for the more expensive drugs, such as fluconazole for stomatitis, 5-HT_3_ antagonists for nausea/vomiting and cytokines for neutropenia. Overall, the mean cost of medication for management of AEs was lower in the capecitabine arm compared with the 5-FU/LV arm (£86 and £345, respectively).

**Figure 2:**
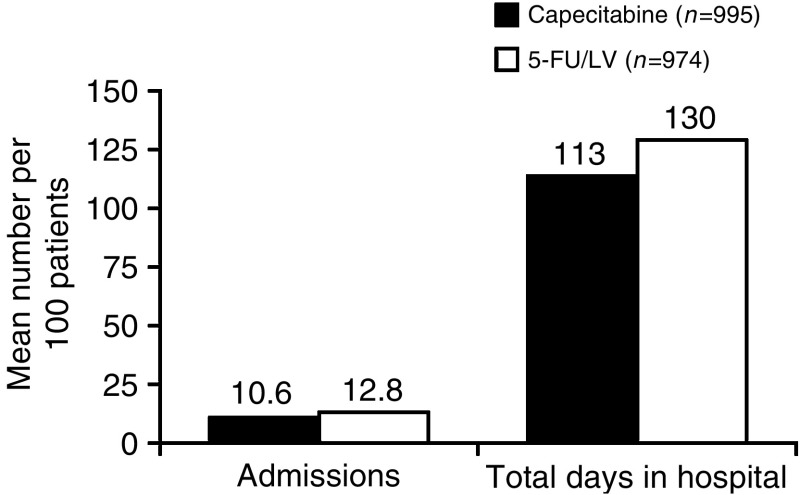
Hospital admissions for AEs.

A similar mean number of physician visits due to AEs were seen in each treatment arm (1.93 and 1.92 for capecitabine and i.v. 5-FU/LV, respectively). However, there were 16% fewer AE-related hospital admissions and 15% fewer days in hospital in the capecitabine treatment arm *vs* the i.v. 5-FU/LV arm (10.6 and 12.8 admissions, respectively, and 113 *vs* 130 days, respectively; Figure 2). The mean cost of hospitalisations was consequently lower with capecitabine than with 5-FU/LV (£399 *vs* £459), although the cost of physician consultations was slightly increased with capecitabine compared with 5-FU/LV (£154 *vs* £145). In accordance with these findings, the projected ambulance costs would be reduced in the capecitabine group compared with the 5-FU/LV group (£38 *vs* £126).

### Societal costs for time and travel

The projected mean number of hours per patient required for travel were lower in the capecitabine group compared with the 5-FU/LV group (27 and 125 h, respectively) and the mean costs for travel time were therefore reduced in the capecitabine group (£320 compared with £1503 in the 5-FU/LV group). Similarly, the mean travel cost per patient was reduced with capecitabine compared with 5-FU/LV (£62 and £196, respectively).

### Total costs

Direct costs during the treatment period have been grouped into six components, as illustrated in Table 3. The major drivers for the cost analysis are the cost of the chemotherapy drugs and the cost of administration of treatment. The additional £4732 required for i.v. therapy is more than three times the additional acquisition cost of capecitabine. With respect to the management of AEs, the most notable difference was the lower cost of medication used for treating AEs in the capecitabine arm (£86 compared with £345 in the 5-FU/LV arm). Overall, from an NHS perspective during the treatment period alone, oral treatment with capecitabine is projected to be cost saving by an average amount of approximately £3653 per patient. From a societal perspective, capecitabine treatment was associated with cost savings of £1184 and £134 for time and travel costs, respectively, yielding cost savings per patient of approximately £4971.

Considering post-treatment costs as well as costs during treatment, the projected direct cost saving for the NHS from a lifetime perspective is projected to be £3608 per patient. From a societal perspective, the lifetime cost savings are even greater: £4925 per patient.

### Clinical effectiveness

**Figure 3:**
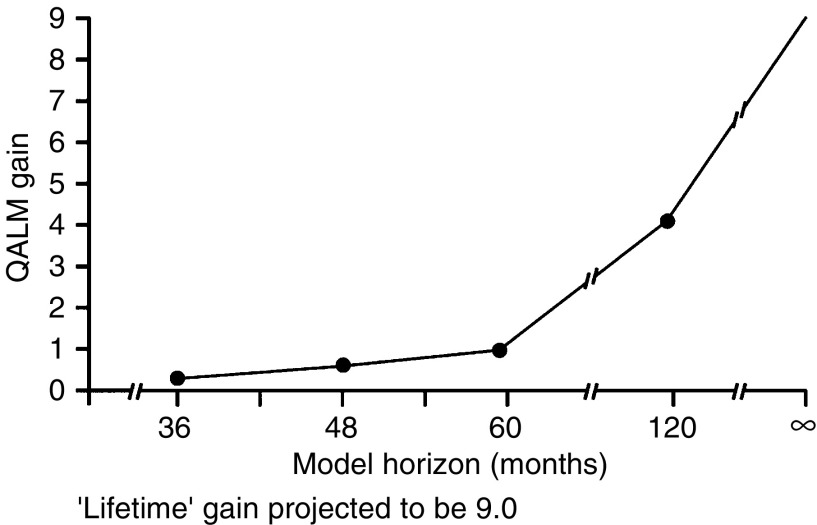
Net gain with capecitabine compared with 5-FU/LV in QALMs by model horizon.

In terms of overall survival, the Kaplan–Meier projection was 81.3% of patients receiving capecitabine surviving at 36 months compared with 77.6% of patients receiving i.v. 5-FU/LV, an absolute difference of 3.7%. In the fitted model, the projected survival gains with capecitabine by 36 and 48 months were 0.5 QALMs and 0.8 QALMs, respectively (Figure 3). When the fitted model is used to extrapolate to longer horizons, for example, 5 years, 10 years or lifetime, the projected gain in QALMs continues to increase with capecitabine, even after taking into account adjustments for quality of life and discounting. Over a lifetime, for example, the QALM advantage for capecitabine widens to 9 months.

### Sensitivity analyses

Table 4 shows the impact of varying model estimates on short-term costs and QALMs. Varying drug acquisition costs for study drugs and medications for management of AEs had only a marginal effect on short-term cost savings: the total cost savings were £14 637 and £14 590 at the 5th and 95th percentiles, respectively. A 20% variation in cost per drug administration visit, however, yielded an almost two-fold variation (£4577–£2707). Overall, the sensitivity analyses confirmed substantial cost savings for oral capecitabine *vs* 5-FU/LV. These analyses also confirmed that the substantial QALM advantage for capecitabine *vs* 5-FU/LV would be maintained even in the face of variation of health state utilities and the discount rate for costs and benefits.

The results of the multi-way sensitivity analysis for post-treatment costs are shown in Table 5. These results demonstrate that the long-term cost advantages of capecitabine are lowest when the costs of relapse and maintenance are low. It is clear that even under rigorous multi-way sensitivity testing, capecitabine remains a robust, cost-saving treatment option compared with 5-FU/LV.

## DISCUSSION

From a UK NHS perspective, this pharmacoeconomic analysis projects that the use of capecitabine for adjuvant treatment of colon cancer would not only save direct medical costs, but also improve health outcomes compared with 5-FU/LV. In economic terms, capecitabine would be termed a ‘dominant’ (cost saving and more effective) treatment strategy, taking its place among other cost-effectiveness benchmarks in oncology (Table 6). The immediate savings on NHS costs during the treatment period with capecitabine would be approximately £3700 per patient. From a societal perspective that also considers patient time and travel costs, the savings would increase to nearly £5000 per patient. In addition, the projected 3.7% absolute improvement in the patient survival outcome observed during the trial period should yield an equivalent of over 9 months of additional survival over a lifetime, after discounting for the time value of money and adjusting for possible quality of life changes due to later relapse.

The key drivers of the dominant cost-effectiveness results of capecitabine in comparison with 5-FU/LV are firstly the savings achieved by avoiding the cost of the i.v. Mayo Clinic regimen for 5-FU/LV, and secondly the projection of improved survival. These are both substantial benefits in comparison to the acquisition cost of capecitabine. The favourable safety profile of capecitabine also translates into lower costs for AEs due to fewer hospitalisations and lower associated medication costs. However, considering costs after the treatment period essentially has a cost neutral impact in the base case: the additional costs of living longer on capecitabine are about the same as the additional costs of earlier and more frequent relapses and death on 5-FU/LV.

In the short term, the critical comparison is between the higher drug acquisition cost of capecitabine (£2081 compared with £602 for 5-FU/LV) and the additional costs for the 28 5-FU/LV infusions (£4732) received by the average patient in the 5-FU/LV treatment arm of the X-ACT trial. We assume that these are provided in an outpatient setting in the UK and that the cost to the NHS is £169 per administration. However, even if the infusion administration costs were as little as one-half of this value, the cost would still be greater than the acquisition cost of capecitabine.

The way in which funding is provided in NHS hospitals, for example, reimbursement for day case attendances, provides some disincentive for them to take a broader NHS perspective, much less the even broader societal perspective. Nonetheless prescribing committees, hospitals and other policy makers should be encouraged to take a broader perspective. Tight prescribing budgets can mean that acquiring approval to switch to capecitabine is difficult but the additional benefits for patients should be weighed in any such decision. Furthermore, although staff costs may be fixed, freeing up their time will allow them to treat more cancer patients quickly and thereby help to reduce waiting lists to government targets.

The other key driver in assessing cost-effectiveness is the projection of improved survival. Although there was only a strong statistical trend towards a survival advantage in the X-ACT trial at 3 years of follow-up, it is important to consider the corroborating evidence. First, the projected survival advantage is reflected in all three, presumably related, measures of disease-free, relapse-free and overall survival (Sargent, 2004). Second, the correlation among these is consistent with previous studies of adjuvant 5-FU/LV in colon cancer. Third, the outcome in the control arm is similar to previous studies using the Mayo Clinic regimen (Haller *et al*, 1998). Furthermore, the strength of the statistical trend was reinforced by the finding that covariate-adjusted survival was significantly superior with capecitabine *vs* 5-FU/LV (hazard ratio 0.788, *P*=0.0208; Twelves *et al*, 2005).

The estimation of the quantitative impact on survival required extrapolation beyond the observed trial period. In similar studies, investigators have approached this in a variety of ways; there is no uniform methodology. We used the approach of fitting a curve to the observed data and extrapolating to the end of life. Both the log-normal and Weibull survival curves are commonly used for this, so both were tried. The fit during the trial period was slightly better for the log-normal curve so this was used in the base case. However, the log-normal distribution yielded a gain of nine QALMs, while the Weibull distribution produced 10.9 QALMs, suggesting that the overall survival results were not sensitive to this choice and were possibly conservative.

The improved survival rates observed with capecitabine, together with the cost savings identified in this and other analyses, render it a viable alternative to 5-FU/LV both as a single agent and in combination. Preliminary phase III data have shown the combination of oxaliplatin and infusional 5-FU/LV (FOLFOX) to be effective in the adjuvant setting (Arkenau *et al*, 2005; Ducreux *et al*, 2005; Sastre *et al*, 2005). Replacing 5-FU/LV with capecitabine in this combination is promising not only clinically, but also economically, as additional infusion and time costs would be avoided.

One limitation of this model is the lack of direct measures of utility in the stable (prerelapse) health state following treatment. Based on the literature, we imputed a relatively high utility value of 0.86 for this health state, which was assumed for both arms. Thus, any impact would be due to the duration of time in this health state, *vs* the time in the postrelapse health state. The postrelapse value was also imputed from the literature to be 0.59, which is similar to the values reported for patients on chronic renal dialysis. Treatment phase utility was assumed to be the same in both arms: 0.80.

The use of a societal perspective to measure the time and travel costs associated with the treatments illustrates the advantage of oral over infusion treatment. On average, patients receiving oral therapy are estimated to spend around 99 fewer hours either receiving treatment or in treatment-related travel. Valued at average market compensation, this amounts to an additional cost saving of about £1300, which is treated here as a cost to society. It could well be the case that many patients would also regard this impact as representing some degree of utility loss with infusion therapy, reflecting a negative impact on their quality of life during the treatment period. The calculations do not take account of such an effect: only the opportunity cost of the time spent is projected.

This pharmacoeconomic analysis found that capecitabine is a dominant (cost saving and more effective) therapy compared with 5-FU/LV from both the NHS and societal perspectives. These results are further supported by other analyses in the Italian healthcare setting, where capecitabine was also found to be cost saving by €2234 per adjuvant treatment (data on file) and in the US, where capecitabine was projected to be a cost-effective therapy from a payer and societal perspective (Garrison *et al*, 2005). Based on these data, the replacement of 5-FU/LV with capecitabine in the adjuvant treatment of colon cancer in the UK would be cost saving and produce better outcomes and hence be strongly cost-effective and preferred.

## Figures and Tables

**Table 1:** Unit cost estimates for medical resource utilisation

	**Capecitabine (£)**	**5FU/LV (£)**
Chemotherapy per g^a^	4.93	382.20

*Visits for drug administration*		
Physician consultation^b^	57	57
i.v. administration visit^c^	0	169

Hospitalisation: cost per day^b^	354	
	
*Provider consultations*		
GP (office)^b^	21	
GP (home visit)^b^	65	
Specialist (office)^b^	57	
Day care^b^	169	
Accident and emergency^b^	83	
Nurse/other office consultation^b^	9	
Nurse/other home visit^b^	20	

Ambulance (round trip)^b^	86	

a Monthly Index of Medical Specialties, September, 2004.

b Netten and Curtis, 2004.

c Chartered Institute of Public Finance and Accountancy, 2005.

**Table 2:** Medications used for management of treatment-related adverse events

	**Days of use per 100 patients**	
**Medication**	**Capecitabine (*n*=995)**	**5-FU/LV (*n*=974)**
Antiemetics/antidiarrhoeals	1933	2534
Dermatologicals/emollients	951	229
Benzodiazapines	152	245
Stomatologicals/triazoles	140	775
Antibiotics/cephalosporins	128	133
Cytokines/growth factors	5	21
Octreotide	8	8

Total	3317	3945

**Table 3:** Results of the cost-effectiveness analysis: costs during treatment

	**Cost impact of capecitabine treatment for colorectal cancer in adjuvant therapy (per patient) £**		
	**Capecitabine**	**5-FU/LV**	**Net cost savings**
*Direct medical costs to the NHS*			
Cost of chemotherapy drugs	2081	602	−1479
Cost of visits for study drug administration	419	5151	4732
Cost of hospital use	399	459	61
Cost of physician consultations for adverse events	154	145	−9
Cost of medication for treating adverse events	86	345	260
Cost of ambulance trips	38	126	88

Subtotal^a^	3176	6829	3653

*Societal costs*			
Cost of time	319	1503	1184
Cost of travel	62	196	134

Total costs^a^	3557	8528	4971

a Numbers may differ because of rounding.

**Table 4:** Results of one-way sensitivity analyses

**Parameter**	**Range**	**Short-term cost savings**	**Lifetime QALMs**
Mean mg of capecitabine use	430 137–414 180	£3614–£3693	No change–No change
Mean mg of 5-FU use	19 820–19 147	£3658–£3649	No change–No change
Mean mg of LV use	973–937	£3660–£3647	No change–No change
Heath state utilities	+20–20%	No change–No change	10.9–6.7
Cost per drug administration visit	+20–20%	£4577–£2707	No change–No change
Discount rate for costs and benefits	3.5%	£3899 (long-term cost savings)	6.5
Total AE medication cost	+20–20%	£3705–£3601	No change–No change
QALMs	Weibull distribution	No change	10.9

**Table 5:** Results of multi-way sensitivity analysis for post-treatment costs

**Post-treatment cost parameters (£)**				
**Prerelapse monthly savings**	**Relapse period**	**Postrelapse monthly maintenance**	**Last year of life**	**Lifetime cost savings (£)**
100 (base case)	25 000	200	10 000	3608
100	10 000	200	5000	2973
200	10 000	400	5000	1813
100	40 000	200	15 000	4242
50	25 000	100	10 000	4185
50	40 000	100	15 000	4819

**Table 6:** Cost-effectiveness benchmarks in oncology

	**Cancer setting**	**Life-expectancy gain (months)**	**Cost per life year gained**
Capecitabine *vs* 5-FU/LV	Colon adjuvant	8.7^a^	Dominant^b^
FOLFIRI *vs* 5-FU/LV^c^	Colorectal metastatic	2.6	£29 000
AT *vs* AC^c^	Breast metastatic	NA	£19 000
CMF *vs* observation^d^	Breast adjuvant	3.6	US$447
Chemotherapy *vs* observation^e^	Breast adjuvant	5.1^a^	US$15 400^a^

NA=not available.

a Quality-adjusted values.

b Cost saving and more effective in terms of quality-adjusted life months.

c National Institute for Health and Clinical Excellence.

d Messori *et al*, 1996.

e Hillner and Smith, 1991.

## Appendix A

The following investigators participated in this study:

Argentina – E Mickiewicz, G Pallotta, E Roca, MS Varela, RC Wainstein

Australia – E Abdi, A Barling, S Begbie, D Bell, R Blum, WI Burns, P de Souza, D Kotasek, J Levi, K Pittman, M Schwarz, C Underhill, D Wyld

Austria – P Balcke, M Baur, D Geissler, P Kier, H Ludwig, K Mach, D Öfner, M Prager, H Steiner

Belgium – J De Grève, D Vanstraelen

Brazil – L Camillo-Coura, G Delgado, S Lago, C Rotstein

Canada – JP Ayoub, O Keller, K Khoo, R Rajan, A Sami, R Wong

Croatia – M Duvnjak, ZK Osijek, R Ostojic, E Vrdoljak

Czech Republic – J Dvorak, J Fínek, I Kocakova, M Kůta, J Nemec, V Svoboda, P Vodvarka

France – FX Caroli-Bosc, G Dabouis, E Gamelin, JL Gaudin, M Giovannini, H Gouerou, JE Kurtz, C Lombard-Bohas, D Peré-Vergé, M Ychou

Germany – W Abenhardt, R Behrens, W Brugger, R Heinze, WD Hirschmann, KW Jauch, E Kettner, B Otremba, H Riess, J Rüschoff, M Schmidt, H Tesch, B Tschechne, M Wolf

Greece – L Boutis, I Katsos, G Panagos

Israel – D Aderka, A Benni, B Klein, A Shani, S Stemmer

Italy – M Airoldi, G Amadori, M Antimi, C Barone, M Bertuccelli, G Biasco, C Bumma, G Comella, P Conte, F Di Costanzo, CM Foggi, V Fosser, S Frustaci, G Gasparini, R Labianca, G Luppi, M Marco, D Mecarocci, A Paccagnella, C Rabbi, S Ricci, A Scanni, V Silingardi, F Smerieri, O Vinante

Latvia – A Brîze, G Purkalne

Poland – M Foszczynska-Kloda

Portugal – P Cortes, B da Costa, J Maurício, E Sanches

Spain – E Aranda, R Cubedo, A Lozano, H Manzano, P Martinez del Prado, R Pérez Carrión, G Pérez-Manga, JJ Valerdi, JJ Valverde, A Velasco

Sweden – G Borghede, H Grönberg, B Gustavson, T Linné, B Lödén, B. Norberg, H Starkhammar, J-H Svensson

Switzerland – M Borner, R Hermann, D Köberle, R Morant, O Pagani, C Sessa, R Stahel

Thailand – S Chakrapee-Sirisuk

United Kingdom – N Bailey, F Daniel, D Dunlop, T Iveson, R James, E Levine, A Makris, A McDonald, L Samuel, M Soukop, W Steward, C Topham

Uruguay – IM Muse

USA – J Eckardt, G Gross, G Justice, L Kalman, R Kerr, CG Leichman, E Levine, V Malhotra, R Pelley, MC Perry, J Posey, M Saleh, J Salvatore, J Wooldridge
